# Supplementary material for: Critical behaviour of the stochastic Wilson-Cowan model
Source: PLoS Comput Biol. 2021 Aug 30;17(8):e1008884. doi: 10.1371/journal.pcbi.1008884 (PMC8432901; doi:10.1371/journal.pcbi.1008884)
Supplement: Attachment — Submitted filename: report_PLoS.pdf [file pcbi.1008884.s001.pdf]

Referee Report

April 2021

The authors consider the issue of criticality and neuronal avalanches in the stochastic Wilson-Cowan (WC) model. It is a very interesting work since the detected critical point with neuronal avalances in the WC model is somewhat new and the paper clarifies several controversies of the literature. I recommend publication in PLoS Comput. Biol. after some revision.

Major concerns

1) The introduction is done by using the parlance of standard equilibrium second order phase transitions (even the Ising model is cited) but the model transition is a "non-equilibrium" "absorbing state transition" in the "Directed Percolation (DP)" universality class. These jargon terms perhaps have not been used in favor of the general audience, but they are very common in the literature and perhaps should be introduced so that the reader can connect more easily with the papers of the References. The description of the WC model as a non-equilibrium system without an Hamiltonian starts only after line 64, occupying a single paragraph. I suggest a bit more of emphasis on the fact that neuronal networks (biological and models) are non-equilibrium systems, that there is a whole field of statistical physics of non-equilibrium phase transitions (perhaps with a citation of a book to aid a newcomer), that there is a whole field of SOC non-equilibrium systems [Bonachela2009][Bonachela2010][Kinouchi2020], because even with the paragraph of the authors, this important point could stay unnoticed to the reader.

2) A doubt: the same model for regular lattices (dimension $d < 4$) would pertain to the branching process (BP) class or to the Compact Directed Percolation (DP) class? At mean-field $d \geq 4$, BP, DP and CDP are all the same? Cowan uses the DP description in:

http://www-sop.inria.fr/manifestations/SemesterCirm/slides/cowan

I do not know if these slides have been published, but if so, the publication should be cited. In these slides, Cowan describes a DP transition, with critical point avalanches. This contrasts with his paper [17] where non critical avalanches are considered, as well observed by the authors.

3) The result $w_c = \beta^{-1}\alpha$ is correct, but gives the impression that we need a fine tuning in a single parameter, the average synaptic weigh w_0. However,

the gain β is also an independent neuronal parameter, so we have not a single critical point but $\beta_c w_c = \alpha$, that is, we have a critical line (hiperbolae) in the w vs β parameter plane. Of course, mathematically, the important variable for the transition is $x = \beta w$ (as $x = \beta J$ in the Ising model). Indeed, in the Ising model, *beta* is chosen as the control parameter and the J is fixed. However, we must remember that w is a synaptic parameter and the gain β is a cell body excitability parameter (in the axonal initial segment (AIS)?), and the two are located differently in the neuron. That is, we have not only a fine tuning, but a non-local fine-tuning, since for criticality the synapses w in the presynaptic cells need information about the postsynaptic β. Would this merit a comment in the paper? Or all of this is irrelevant?

4) The (Cowan?) notation Σ_0, w_0, R_0 and s_0 is somewhat confusing to me because it suggests that these quantities refer to the zero activity (absorbing) phase. Also, the fixed points $\Sigma_0 = 0$ and the non zero $\Sigma_0 > 0$ receive the same name in the Methods section (and also in lines 125-126), which is a bit confusing because both exist above w_{0c}: one is the $\Sigma_0 = 0$ which is a true fixed point, but unstable, and the another is $\Sigma_0 > 0$, that is stable, as usual in transcritical bifurcations. Although not used by Cowan [17], I suggest, to aid the readers, the notation Σ_+, R_+ and s_+ or other notation for the stable non-zero solution, reserving $\Sigma_0 = 0$ for the absorbing phase (that also exists above w_c as an unstable fixed point). I also do not understand why to use w_0 in the definition of line 107, since w_0 is not a fixed point but a parameter and we could use $w = w_E - w_I$ and $\Sigma^* = (w - w_c)/w$ which is much more clean. Or the single letter w has been used before? Please, could you clarify the option for this notation? Is it only to preserve the notation of reference [17]?

Minor concerns

6)There is a typo in Eq. (1): it is $\sum_j$ instead of $\sum_{ij}$.

7) A doubt: in Eq. (2), is the correct form $\beta \tanh(s)$ or $\tanh(\beta s)$? I ask because Eq (4) from [17] has no beta, and it admits a beta inside the tanh(), as usual in other stochastic models, for example Gerstner model, Wulfram Gerstner and J Leo van Hemmen (1992) Associative memory in a network of 'spiking' neurons, Network: Computation in Neural Systems, 3:2, 139-164, and Boltzmann Machines. Or this is irrelevant due to the linear approximation?

8) It is not very clear where the authors change from the single neuron s_i representation to the population representation s. Could the authors point better that to the reader?

9) Only a suggestion: in statistical physics it seems more common to define avalanche critical exponents as positive numbers, that is, $P(S) = S^{-\tau_S}$ instead of S^{τ_S}, so that $\tau_S = 3/2$ is positive. The minus sign also already signalizes that $P(S)$ is a decreasing function of S, the reader need not think very much about that. But if the authors opt for negative exponents, then this must be corrected in Fig. 4e, where the exponents are given as positive, and also in the legend of Fig. 5.

10) In line 16, the three citations are from the same group and about the same self-organizing mechanism. Perhaps a very recent review of self-organizing mechanisms could be useful here [Kinouchi2020]. Of course, this is only a suggestion.

Observation about the limit $h \to 0^+$: The fine tuning of $h = 0$ is a welcome emphasis of this paper, because it is poorly discussed in the neuronal avalanches literature. So, in the model, we must fine tune (or self-organize) two independent parameters, w and h. Although $h = 0$ is natural for magnetic spin systems, it is not so natural to neuronal networks where neurons are always bombarded with inputs external to the network. However, there exist an idea to obtain $h = 0$ (this explanation need not be included in the paper, it is only a clarification for the authors). The general form of Eq. (1) is $s_i = \sum_j w_{ij} a_j + I_i - \theta_i$, here I_i are external inputs and θ_i are the neuron firing thresholds (or biases). Now, suppose that the thresholds are adaptive so that their dynamics $\theta_i(t)$ tend to cancel the inputs I_i, as occur in perfect sensory adaptation and perfect firing rate adaptation. Then, although the biological external inputs are not vanishing, the field $h = I - \theta$ can be very close to zero, where $I = \langle I_i \rangle$ and $\theta = \langle \theta_i \rangle$. This self-organization of $\theta_i(t)$ to obtain a fixed point $h^* \approx 0$ has been proposed recently in [Girardi2020,Kinouchi2020] and the level $h = 10^{-6}$ or less can be easily achieved in these models.

Suggestion for future work, not for this paper: I think that if you use the function $f(s) = \beta s/(1 + \beta s)$ instead of $f(s) = \beta \tanh(s)$, a lot of exact, instead of approximate, results can be obtained. In particular, an equation similar to Eq. (11) is exact (with the difference of some factors 2): $2\beta w \Sigma^{*2} + (\alpha - \beta w + 2\beta h)\sigma^* - \beta h = 0$. Since this equation is exact, $\Sigma_+ = (w - w_c)/w$ is valid for all (even large) w and is not a first order approximation for this particular $f(s)$.

References

I am not requiring that the authors cite these references, only that they consider if they are useful.

[Bonachela2009] Bonachela, J. A. and Munoz, M. A. Self-organization without conservation: true or just apparent scale-invariance?. Journal of Statistical Mechanics: **2009**, P09009 (2009).

[Bonachela2010] Bonachela, J. A., De Franciscis, S., Torres, J. J. and Munoz, M. A. Self-organization without conservation: are neuronal avalanches generically critical?. Journal of Statistical Mechanics: **2010**, P02015 (2010).

[Girardi2020] Girardi-Schappo, M., Brochini, L., Costa, A. A., Carvalho, T. T. A. and Kinouchi, O. Synaptic balance due to homeostatically self-organized quasicritical dynamics. Physical Review Research: **2**,012042 (2020).

[Kinouchi2020] Kinouchi, O., Pazzini, R. and Copelli, M. Mechanisms of Self-Organized Quasicriticality in Neuronal Network Models. Frontiers in Physics: **8**, 530 (2020).
