# Supplementary material for: Critical behaviour of the stochastic Wilson-Cowan model
Source: PLoS Comput Biol. 2021 Aug 30;17(8):e1008884. doi: 10.1371/journal.pcbi.1008884 (PMC8432901; doi:10.1371/journal.pcbi.1008884)
Supplement: Attachment — Submitted filename: avalrep.pdf [file pcbi.1008884.s002.pdf]

Report on "Critical Behaviour of the Wilson-Cowan Model" by de Candia et al.

This paper reports on critical behavior at a "second-order-like" phase transition in the stochastic Wilson-Cowan (SWC) model for a microscopic network of individual neurons. It finds critical exponents for avalanches that match those seen in experiments at these scales.

For the reasons listed below, the paper is not acceptable in anything like its present form, so I must recommend rejection.

Major Comments.

1. This paper has some quite interesting abstract results, but it needs to link applications of the population-level WC model, and more modern models in the same family, which find a **first-order** phase transition at larger scales (e.g, works by Steyn Ross, Wilson, Breakspear, Robinson, and others over the last 25 years). One wonders how the "2nd-order-like" phase transition at the scales simulated here relates to these findings at larger scales, especially as the two size ranges overlap (see below). This point needs to be resolved.
2. Links to the literature on brain stability and criticality are very poor (e.g., as mentioned in the previous point). There is a lot more to this field than the avalanche literature that stems directly from Beggs and Plenz's paper, and much of it predates that work. E.g., on p.5, lines 121ff, there are no references to the extensive studies of stability and power-law spatiotemporal spectra of similar equations made 10-40 years back by numerous authors including the above plus Freeman, Nunez, Jirsa, and many others. The present authors need to clarify which of their results are new and which are are actually in the (uncited here) literature.
3. The paper needs to stress that many of the simulations only apply to sub-mm scales. For example, 10000 neurons would underlie only a 0.3 mm square of cortical surface. On the other hand the larger simulations of 10^7 neurons would correspond to about a 10 mm square, which overlap with the macroscopic WC regime. Hence, it is imperative to resolve the prima facie contradiction with the 1st order transition seen in macroscopic WC models which also have the advantage that they have quantitatively explained a wide range of other phenomena – alpha and other rhythms, EEG spectra, correlations, seizures, evoked responses, etc. If your model appears to contradict an experimentally verified model with wider applicability the onus is on you to explain what is going on – either to resolve a paradox or explain where the other models are wrong, despite being experimentally verified.
4. What other phenomena does the present model explain? In general, a theory of multiple phenomena is to be preferred to one that is purpose built for a single application but has many free parameters, especially when the sensitivity to these parameters is not fully explored.
5. The all-to-all coupling of the neurons, introduced on p3, is unrealistic, even at short scales, and certainly at N>10^4 because there are only about 10^4 synapses per neuron – so the greatest possible connectivity probability is 0.001 (not 1.0!) in the largest simulations considered. What effect would a more realistic connectivity level, with a roughly exponential range distribution, yield? The claim that this work relates to realistic brain dynamics should be toned down and it should be acknowledged that this is a toy model. Perhaps it would be better submitted to a statistical physics journal? Overall, I suspect that many of the key results (e.g., firing rate rising with N and quite probably the class of critical point) are the product of this unphysical assumption, but it is impossible to know for sure without this point being explored. The all-to-all

assumption needs to be highlighted in the abstract and its unrealistic nature needs to be treated in the discussion.

6. Steyn Ross and Wilson have used similar stochastic methods to study WC-like equations at larger scales, finding a first-order phase transition. Again, the links need to be made explicit.
7. The claims in the Discussion are overstated – the best one can say from this analysis is that the SWC model results are qualitatively similar to some seen in neural systems. It also needs to be acknowledged that the present work contradicts larger-scale WC-family results where the latter have been experimentally verified at scales of mm and above.

Other points:

8. It would be good to highlight in a fig like fig 1a where the system is operating on the effective (smoothed) firing rate response curve. The implication is that it is very close to 0.1 on the horizontal axis. Actual mean firing rates of real cortical neurons (5-10 per second) are much less than maximum rates (a few hundred per second), which normally would place them to the left of this point in Fig 1a. I note though that the maximum possible firing rate is not clear from this figure – it would be good to mention it – so this comment is subject to that proviso.
9. Please clarify the units of s, h, w,f, etc. If they are dimensionless, the way in which they have been nondimensionalized needs to be made explicit. Are the numerical values consistent with independent measurements of these quantities? A referenced table of the assumed values of all quantities would be useful.
10. How robust are the results with respect to changes in parameters? This needs to be explored to reassure the reader that the results are more than a fluke.
11. Real cortical neurons receive around 10^5 spikes per second (10 per second via 10^4 synapses), so they produce 1 output spike for roughly each M=1000 input spikes for alpha = 0.1/ms (10 ms integration time). Hence, the input signal is essentially continuous, with some fluctuations, not spiky. Many spiking neuron simulations in the literature do not satisfy the requirement M>>1. Please evaluate and mention.

There are many other minor comments that could be added, but the central issues suffice to support a recommendation that the paper be rejected as-is. It should either be submitted to a statistical mechanics journal, without the claims that it is a realistic representation of brain tissue, or rewritten and extended to address the above issues before submission as a new paper. In either case, proper referencing of the prior literature is essential.
