# Supplementary material for: Critical behaviour of the stochastic Wilson-Cowan model
Source: PLoS Comput Biol. 2021 Aug 30;17(8):e1008884. doi: 10.1371/journal.pcbi.1008884 (PMC8432901; doi:10.1371/journal.pcbi.1008884)
Supplement: Attachment — Submitted filename: response.pdf [file pcbi.1008884.s003.pdf]

Response to Report of Referee 1

We thank the referee for writing that our study "is a very interesting work since the detected critical point with neuronal avalanches in the WC model is somewhat new and the paper clarifies several controversies of the literature" and for recommending publication. In the following we address all comments raised by the referee.

Major concerns

1) The referee writes:

The introduction is done by using the parlance of standard equilibrium second order phase transitions (even the Ising model is cited) but the model transition is a "non-equilibrium" "absorbing state transition" in the "Directed Percolation (DP)" universality class. These jargon terms perhaps have not been used in favor of the general audience, but they are very common in the literature and perhaps should be introduced so that the reader can connect more easily with the papers of the References. The description of the WC model as a non-equilibrium system without a Hamiltonian starts only after line 64, occupying a single paragraph. I suggest a bit more of emphasis on the fact that neuronal networks (biological and models) are non-equilibrium systems, that there is a whole field of statistical physics of non-equilibrium phase transitions (perhaps with a citation of a book to aid a newcomer), that there is a whole field of SOC non-equilibrium systems [Bonachela2009][Bonachela2010][Kinouchi2020], because even with the paragraph of the authors, this important point could stay unnoticed to the reader.

We thank the referee for the interesting comment. We fully agree with the observation that neuronal systems, biological systems in general, are out of equilibrium and therefore the description is much more complex than a simple order-disorder transition. The reason why we wrote an extensive paragraph on equilibrium phase transition is because in our opinion there is some confusion in the literature about the conditions under which a second order phase transition can be observed. The specific case concerns the previous results of ref.17 (ref. 26 in the revised version), concluding that the WC model does not have a critical avalanche behavior but neglecting that simulations were performed in non-zero external field and not precisely at the critical point. Since we fully agree with the referee, we have extended the paragraph on non-equilibrium systems and we have added the references mentioned by the referee.

In the revised version we have added the paragraph at lines 85-100.

We have also included the new references Bonachela2009, Bonachela2010, Kinouchi2020.

2) The referee writes:

A doubt: the same model for regular lattices (dimension d < 4) would pertain to the branching process (BP) class or to the Compact Directed Percolation (DP) class? At mean-field d >= 4, BP, DP and CDP are all the same? Cowan uses the DP description in:

http://www-sop.inria.fr/manifestations/SemesterCirm/slides/cowan

I do not know if these slides have been published, but if so, the publication should be cited. In these slides, Cowan describes a DP transition, with critical point avalanches. This contrasts with his paper [17] where non critical avalanches are considered, as well observed by the authors.

We thank the referee for raising this interesting question. Indeed, this was longtime an intriguing point also for us. How is it possible to observe for avalanche activity mean field exponents in finite dimensions? Experimental data in 2d and 3d systems indeed confirm the mean field values independently of system dimensionality. We first comment that both directed percolation and branching process on a tree, therefore in the mean field approximation, do provide the same universality class. Both characterizations are therefore equivalent on a tree. In CDP clusters are compact and, to our knowledge, CDP is not usually implemented on a tree. According to the DP-conjecture by Janssen and Grassberger, systems with short-range interactions, exhibit a continuous phase transition into a single absorbing state, belonging generically to the DP universality class, provided that they are characterized by a one component order parameter without additional symmetries and without unconventional features such as quenched disorder. Non-DP

behavior is expected to occur in systems where at least one of these requirements is not fulfilled. We follow the suggestion by the referee and concerning these aspects of non-equilibrium phase transitions we now cite the book “Non-Equilibrium Phase Transitions” by Malte Henkel, Haye Hinrichsen and Sven Lübeck (Springer Netherlands 2008).

The crucial issue is how is possible to observe mean field behavior in real systems. One possible answer is the small world feature of functional networks, well supported by a number of experimental results. Moreover, in a recent manuscript some of us (von Kessenich et al, Sci Rep 2016) showed that, starting from a regular square lattice, a neuronal integrate and fire model shows a crossover from the 2d sand pile behavior to the mean field branching process universality class. This change in behavior is due to the interplay between synaptic plastic adaptation and refractory time which makes the regular lattice evolve into a tree with negligible loops.
In order to clarify this point in the context of the WC model we performed additional simulations on a 2d square lattice 32x32.On each site of the lattice are placed one excitatory and one inhibitory neuron, for a total number of neurons N=2048. Each neuron can establish an average number of 80 synaptic connections at random, and the connection probability is proportional to exp(-r/5), where r is the distance between two neurons. Preliminary results show that the avalanche activity exhibits exponents very close to the values detected for the fully connected network, as shown in the attached figure. However, due to the limited system size the scaling regime is slightly larger than one decade and the estimate of the exponents is not fully accurate. Moreover, in this calculation we implemented the value of the critical point w_0c found for the fully connected network, even if a more accurate identification is necessary since the critical point is not a universal quantity but depends on the network structure. Therefore, we plan to investigate the WC behavior in finite dimensions in more detail and with a better statistics in the next future.
We did not find notice of publication for the Cowan slides. They are the presentation made by Cowan in the INRIA center in Nice. We have no problem in including them in the reference list if the journal allows.
In the revised version we have added the comment on the WC model behavior in finite dimension in the Discussion, at lines 451-475, and the new references von Kessenich et al, Sci Rep, 2016 and the book by Henkel et al.

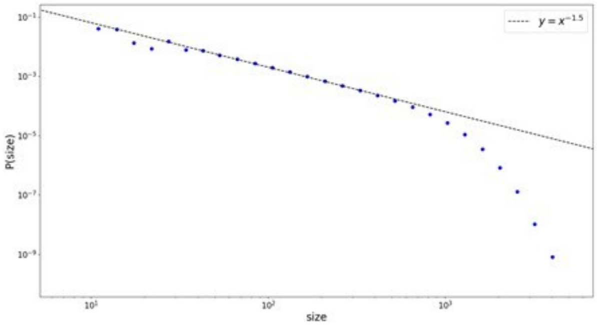

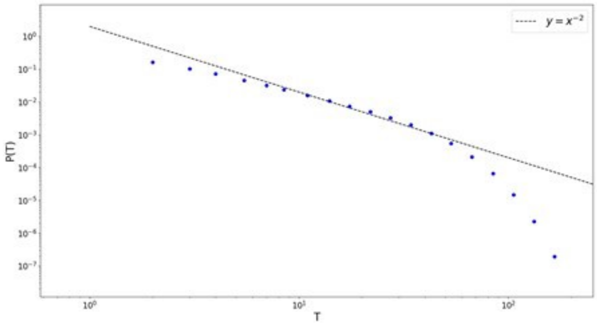

3) The referee writes:
The result wc =beta^-1 alpha is correct, but gives the impression that we need a fine tuning in a single parameter, the average synaptic weigh w0. However, the gain beta is also an independent neuronal parameter, so we have not a single critical point but beta_cwc = alpha, that is, we have a critical line (hiperbolae) in the w vs beta parameter plane. Of course, mathematically, the important variable for the transition is x = betaw (as x = betaJ in the Ising model). Indeed, in the Ising model, beta is chosen as the control parameter and the J is fixed. However, we must remember that w is a synaptic parameter and the gain beta is a cell body excitability parameter (in the axonal initial segment (AIS)?), and the two are located differently in the neuron. That is, we have not only a fine tuning, but a non-local fine-tuning, since for criticality the synapses w in the presynaptic cells need information about the postsynaptic beta. Would this merit a comment in the paper? Or all of this is irrelevant?

We thank the referee for his wise insights that are far from being irrelevant and are rather very clever and stimulating in the direction of clarifying the analogy with second order phase transitions. Indeed, the impression the referee had is correct and is the direct consequence of a crucial property of neuronal systems. The tuning of a single parameter is sufficient since it is the expression of the so-called balance of excitation and inhibition characterizing healthy behavior in neuronal systems. Indeed, breaking such balance leads to pathological (supercritical) behavior typical of epileptic systems. This balance is imbedded in the WC model, as testified by the fixed point solution Delta=0. Our study of the critical point confirms the crucial role of balance since alpha represents the dis-activation rate, conversely beta the activation one. Their ratio is therefore a measure of the activity – quiescence balance at the level of a single neuron. The existence of a critical ratio value (w_c), to be associated to critical (and therefore healthy) behavior is a further confirmation of excitation-inhibition balance. Somehow this is also reminiscent of the balance of the energetic and entropic contributions leading to the minimum of the free energy in physical systems. Concerning the non-locality of self-tuning, it is certainly true that such non-local balance is at work in biological systems and certainly represents another striking feature of living neuronal networks.
In the revised version we have commented on non-local self-tuning in the Discussion section, at lines 381-393.

4) The referee writes:
The (Cowan?) notation Sigma0; w0; R0 and s0 is somewhat confusing to me because it suggests that these quantities refer to the zero activity (absorbing) phase. Also, the fixed points Sigma0 = 0 and the non zero Sigma0 > 0 receive the same name in the Methods section (and also in lines 125-126), which is a bit confusing because both exist above w0c: one is the Sigma0 = 0 which is a true fixed point, but unstable, and the another is Sigma0 > 0, that is stable, as usual in transcritical bifurcations. Although not used by Cowan [17], I suggest, to aid the readers, the notation Sigma+;R+ and s+ or other notation for the stable non-zero solution, reserving Sigma0 = 0 for the absorbing phase (that also exists above wc as an unstable fixed point). I also do not understand why to use w0 in the definition of line 107, since w0 is not a fixed point but a parameter and we could use w = wE - wI and Sigma = (w-wc)/w which is much more clean. Or the single letter w has been used before? Please, could you clarify the option for this notation? Is it only to preserve the notation of reference [17]?*

We implemented the same notation of ref.17 (ref. 26 in the revised version) in the attempt to make the manuscript more accessible to readers knowing the previous papers by Cowan. The zero index in this quantity just refers to the fixed point solution, regardless if it is zero or nonzero. Since this notation has been kept in a number of well cited manuscripts, it is familiar to readers. For this reason, we hope that the referee will understand if we prefer not to change it introducing novel definitions, since it could cause confusion in the readership.

Minor concerns

6)*There is a typo in Eq. (1): it is Sum_j instead of Sum_ij*
We thank the referee for noticing the typo. **We corrected it in the revised version**.

7) *A doubt: in Eq. (2), is the correct form beta tanh(s) or tanh(beta s)? I ask because Eq (4) from [17] has no beta, and it admits a beta inside the tanh(), as usual in other stochastic models, for example Gerstner model, Wulfram Gerstner and J Leo van Hemmen (1992) Associative memory in a network of `spiking' neurons, Network: Computation in Neural Systems, 3:2, 139-164, and Boltzmann Machines. Or this is irrelevant due to the linear approximation?*
The beta factor is introduced for dimensional reasons since f(s) represents a firing rate and therefore must have the dimension of an inverse time. The correct expression is therefore beta tanh(s). Interestingly this factor, missing in ref.17 (ref. 26 in the revised version) as the referee rightly noticed but present in successive articles by the same authors, plays an important role in determining the critical point.

8) *It is not very clear where the authors change from the single neuron si representation to the population representation s. Could the authors point better that to the reader?*

The explanation is shortly summarized after the equations (4) that are expressed in terms of the number of active excitatory and inhibitory neurons. This change from single neuron to the two-component population has been first introduced by Wilson and Cowan in 1972 and then reported in all papers studying this model. Since the large size expansion and the linear noise approximation are not novel and require many details, we decided to address the readers to ref.17 which provides all calculations in detail.
In the revised version we have introduced a new citation to ref.17 (ref. 26 in the revised version) just before Eq.3.

9) *Only a suggestion: in statistical physics it seems more common to define avalanche critical exponents as positive numbers, that is, P(S) = S^-tauS instead of S^tauS , so that tauS = 3/2 is positive. The minus sign also already signalizes that P(S) is a decreasing function of S, the reader need not think very much about that. But if the authors opt for negative exponents, then this must be corrected in Fig. 4e, where the exponents are given as positive, and also in the legend of Fig. 5.*
We understand the point raised by the referee. **In the revised version we have changed the exponent notation and refer to their positive values.**

10) *In line 16, the three citations are from the same group and about the same self-organizing mechanism. Perhaps a very recent review of self-organizing mechanisms could be useful here [Kinouchi2020]. Of course, this is only a suggestion.*
In the revised version we have added the reference suggested by the referee, at line 19.

Observation about the limit h -> 0+: *The fine tuning of h = 0 is a welcome emphasis of this paper, because it is poorly discussed in the neuronal avalanches literature. So, in the model, we must fine tune (or self-organize) two independent parameters, w and h. Although h = 0 is natural for magnetic spin systems, it is not so natural to neuronal networks where neurons are always bombarded with inputs external to the network. However, there exist an idea to obtain h = 0 (this explanation need not be included in the paper, it is only a clarification for the authors). The general form of Eq. (1) is si =Sum_j wijaj +Ii -thetai, here Ii are external inputs and thetai are the neuron firing thresholds (or biases). Now, suppose that the thresholds are adaptive so that their dynamics_i(t) tend to cancel the inputs Ii, as occur in perfect sensory adaptation and perfect _ring rate adaptation. Then, although the biological external inputs are not vanishing, the field h = I -theta can be very close to zero, where I = <I> and theta= <theta>. This self-organization of thetai(t) to obtain a fixed point h_ _ 0 has been proposed recently in [Girardi2020,Kinouchi2020] and the level h = 10[illegible]6 or less can be easily achieved in these models.*
Another very interesting remark based on recent manuscripts we know. We will address the possibility of criticality in non-zero field by self adaptation in the next developments of this study.
In the revised version we have added both references suggested by the referee, at line 373.

Suggestion for future work, not for this paper: *I think that if you use the function f(s) = beta s=(1 + betas) instead of f(s) = beta tanh(s), a lot of exact, instead of approximate, results can be obtained. In particular, an equation similar to Eq. (11) is exact (with the difference of some factors 2): 2_w__2 +(_ [illegible] _w + 2_h)__ [illegible] _h = 0. Since this equation is exact, _+ = (w [illegible] wc)=w is valid for all (even large) w and is not a first order approximation for this particular f(s).*
We thank the referee for this interesting suggestion. We will certainly consider this different activation function in the next developments of this study.

References
I am not requiring that the authors cite these references, only that they consider if they are useful.

[Bonachela2009] Bonachela, J. A. and Munoz, M. A. Self-organization without conservation: true or just apparent scale-invariance?. Journal of Statistical Mechanics: 2009, P09009 (2009).

[Bonachela2010] Bonachela, J. A., De Franciscis, S., Torres, J. J. and Munoz, M. A. Self-organization without conservation: are neuronal avalanches generically critical?. Journal of Statistical Mechanics: 2010, P02015 (2010).
[Girardi2020] Girardi-Schappo, M., Brochini, L., Costa, A. A., Carvalho, T. T. A. and Kinouchi, O. Synaptic balance due to homeostatically self-organized quasicritical dynamics. Physical Review Research: 2,012042 (2020).
[Kinouchi2020] Kinouchi, O., Pazzini, R. and Copelli, M. Mechanisms of Self-Organized Quasicriticality in Neuronal Network Models. Frontiers in Physics: 8, 530 (2020).
In the revised version we have included all these references.

Response to Report of Referee 2

In the following we answer to all objections raised by the referee.

Major Comments.
1. This paper has some quite interesting abstract results, but it needs to link applications of the population-level WC model, and more modern models in the same family, which find a ***first-order*** *phase transition at larger scales (e.g, works by Steyn Ross, Wilson, Breakspear, Robinson, and others over the last 25 years). One wonders how the "2nd-order-like" phase transition at the scales simulated here relates to these findings at larger scales, especially as the two size ranges overlap (see below). This point needs to be resolved.*

We really thank the Referee for raising this important point, which allows us to better frame our study in a more general context within the modeling of brain activity. Concerning the papers mentioned by the referee, a general comment is that they treat cases that are beyond our scope. Our study focuses on the spontaneous activity, namely the activity of an alive system in the absence of any external stimulation and in healthy conditions. More precisely, we stress that we consider the Wilson-Cowan model in the limit of vanishing external field, $h \rightarrow 0$ in the activation function, a condition which puts our results in a completely different regime with respect to that studied, for instance, in Negahbani, Steyn-Ross et al. J. Math. Neurosci. 5:9 (2015). Here, the authors considered the bifurcation transitions appearing upon varying the (always finite) external voltage inputs. The observed first-order transitions therefore occur in a region of the parameter space of the model which does not overlap with that studied in our work. As an example, we mention that the Ising model exhibits a second order phase transition at the critical temperature in zero field, however it also shows a first order phase transition for varying non-zero magnetic fields. The choice of the control parameters is therefore crucial in determining the order of the phase transition and the two phenomena can coexist in the same model. Moreover, the theoretical results by these authors show slowing down and increasing fluctuations close to the bifurcation point and describe real systems in specific conditions, such as anesthesia, sleep cycles or seizures (as for instance described in "Phase transitions in single neurons and neural populations: Critical slowing, anesthesia, and sleep cycles" D.A. Steyn-Ross, M.L. Steyn-Ross, M.T. Wilson, and J.W. Sleigh, Chapter 1 of the book "Modeling phase transitions in the brain"). In the context of critical phenomena, slowing down and increase in fluctuations are considered critical if the relaxation time and the fluctuations diverge with the system size. This is the signature of scale invariance, a necessary ingredient for a system to be at a critical point. Our use of the term "critical" is to be understood in this theoretical context.

Since our study does not apply to the case of strong external stimulations, such as administration of anesthetic drugs, neither to the case of transitions between different sleep states, neither to the situation where epileptic crises can occur, there is no contradiction between the behaviors described by these authors and those considered by us: They fall in different classes and describe different phenomena. In our case, the parameter driving the transition is not the external input (which is kept as small as possible), but

rather the unbalance of the synaptic weight w_0 between excitatory and inhibitory neuron populations. Among the references suggested by the Referee, in the review by Breakspear and co-workers (Cocchi et al. Progress in Neurobiology 158, 132 (2017)) the possible different behaviors in brain activity are discussed. In that paper, it is shown how the thermodynamic phase transitions in spatially extended systems are somehow the counterpart of the bifurcation transitions for systems with few components. This is an interesting point of view, however our considerations apply to systems with a sufficiently large number of degrees of freedom, namely in the thermodynamic limit.

The main conclusion of our study is that the stochastic Wilson-Cowan model, in the limit of zero external field, shows a second-order transition, with an order parameter passing continuously from zero to a finite value, and with a power-law scaling of the avalanche distributions. This claim does not contradict previous results obtained in different regions of the parameter space.

In the revised version of the manuscript we devoted a new paragraph in the Discussion to address in detail the differences with respect to the previous studies mentioned by the Referee, in order to provide the reader with a wider overview of the possible phenomenology, at lines 351-368. We also added the new references [36,44,45,46,47].

2. Links to the literature on brain stability and criticality are very poor (e.g., as mentioned in the previous point). There is a lot more to this field than the avalanche literature that stems directly from Beggs and Plenz's paper, and much of it predates that work. E.g., on p.5, lines 121ff, there are no references to the extensive studies of stability and power-law spatiotemporal spectra of similar equations made 10-40 years back by numerous authors including the above plus Freeman, Nunez, Jirsa, and many others. The present authors need to clarify which of their results are new and which are actually in the (uncited here) literature.

We thank the Referee for pointing out other previous works on stability and power-law behaviors of brain activity. It is true that we mainly focused on literature concerning avalanche dynamics and the Wilson Cowan model. We understand the point raised by the referee and, following his/her suggestion, we have added a novel paragraph in the introduction, summarizing these main results. Concerning our results, we were inspired by the manuscript ref.17 (ref. 26 in the revised version), however all our results are new and enlighten properties of the WC model not discussed in that paper.

In the revised version of the manuscript we have added a new paragraph in the beginning of the introduction, at lines 2-5, with additional references to the works mentioned by the referee (Refs.[1-8]).

3. The paper needs to stress that many of the simulations only apply to sub-mm scales. For example, 10000 neurons would underlie only a 0.3 mm square of cortical surface. On the other hand the larger simulations of 10^7 neurons would correspond to about a 10 mm square, which overlap with the macroscopic WC regime. Hence, it is imperative to resolve the prima facie contradiction with the 1st order transition seen in macroscopic WC models which also have the advantage that they have quantitatively explained a wide range of other phenomena – alpha and other rhythms, EEG spectra, correlations, seizures, evoked responses, etc. If your model appears to contradict an experimentally verified model with wider applicability the onus is on you to explain what is going on – either to resolve a paradox or explain where the other models are wrong, despite being experimentally verified.

We thank the referee for this remark and we wish to point out that one of the fundamental properties of a system sitting at the critical point is scale invariance: The properties of the system exhibit the same behaviour at different scales. Indeed, the statistics of avalanches shows consistently similar behaviour from LFP data for dissociated neurons to MEG data for the entire brain. Moreover, as already discussed in the previous point, our results do not contradict previous studies of the same model. They account for different phenomena observed in spontaneous activity of healthy systems, in the absence of external stimuli. In particular, we do not claim that our results apply to stimulated activity or seizures. These behaviors are observed for a different tuning of parameters.

4. What other phenomena does the present model explain? In general, a theory of multiple phenomena is to be preferred to one that is purpose built for a single application but has many free parameters, especially when the sensitivity to these parameters is not fully explored.

In the present manuscript we do not introduce a novel model but we study the stochastic WC model which has received a wide attention in the literature and it has been shown to exhibit different behaviours, as oscillations, noisy limit cycles, quasi cycles, avalanches, etc… We are interested in showing that the phenomenology presented by the stochastic WC model in a certain region of the parameter space is in agreement with an interpretation in the framework of critical phenomena (in the sense of second-order phase transitions). Our claim is in contrast with previous studies where the avalanche behaviors was simply ascribed to the nonlinear nature of the model [ref. 17 by Benayoun et al]. Our study explores a wider range of parameters with respect to that work and illustrates the sensitivity of the model to these parameters. In particular we identified a critical value for the control parameter and focused on values around this critical point. Our study clarifies the results by ref.17 showing how the apparent lack of critical behaviour is due to the departure from the critical behavior and to the presence of a non-vanishing field. Concerning the range of variation of parameters, we vary the system size (from 10^3 to 10^7), the unbalance in synaptic strengths (from 0 to 1) and the external field (from 10^-3 to 10^-6). All other parameters are not relevant to determine the critical behaviour.

5. The all-to-all coupling of the neurons, introduced on p3, is unrealistic, even at short scales, and certainly at N>10^4 because there are only about 10^4 synapses per neuron – so the greatest possible connectivity probability is 0.001 (not 1.0!) in the largest simulations considered. What effect would a more realistic connectivity level, with a roughly exponential range distribution, yield? The claim that this work relates to realistic brain dynamics should be toned down and it should be acknowledged that this is a toy model. Perhaps it would be better submitted to a statistical physics journal? Overall, I suspect that many of the key results (e.g., firing rate rising with N and quite probably the class of critical point) are the product of this unphysical assumption, but it is impossible to know for sure without this point being explored. The all-to-all assumption needs to be highlighted in the abstract and its unrealistic nature needs to be treated in the discussion.

We wish to point out that most of the articles on the WC model focus on the all to all connectivity. In particular, results for avalanche dynamics in ref.17 were obtained with this fully connected network. In this reference authors also analyse the case of sparse networks, evidencing the presence of avalanche dynamics also in sparse systems.

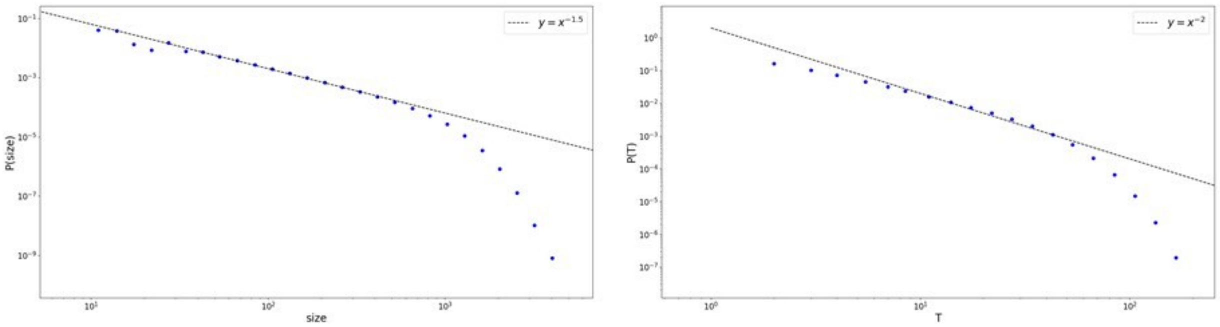

However, we understand the point raised by the referee and we have produced numerical results for the WC model on a 2d square lattice 32x32. On each site of the lattice are placed one excitatory and one inhibitory neuron, for a total number of neurons N=2048. Each neuron can establish an average number of 80 synaptic connections at random, and the connection probability is proportional to exp(-r/5), where r is the distance between two neurons. Preliminary results show that the avalanche activity exhibits exponents very close to the values detected for the fully connected network, as shown in the attached figure.

However, due to the limited system size the scaling regime is slightly larger than one decade and the estimate of the exponents is not fully accurate. Moreover, in this calculation we implemented the value of the critical point w_0c found for the fully connected network, even if a more accurate identification is necessary since the critical point is not a universal quantity but depends on the network structure. Therefore, we plan to investigate the WC behavior in finite dimensions in more detail and with a better statistics in the next future

In the revised version we have added the comment on the WC model behavior in finite dimension in the Discussion, at lines 451-475.

6. Steyn Ross and Wilson have used similar stochastic methods to study WC-like equations at larger scales, finding a first-order phase transition. Again, the links need to be made explicit.

Please, see our response to point 1.

In the revised version we discuss this point in a novel paragraph in the Discussion, at lines 351-368.

7. The claims in the Discussion are overstated – the best one can say from this analysis is that the SWC model results are qualitatively similar to some seen in neural systems. It also needs to be acknowledged that the present work contradicts larger-scale WC-family results where the latter have been experimentally verified at scales of mm and above.

This question raises again concerns expressed in points 1 and 3. We believe that the different behaviour is not due to the system size but rather to the different region of parameters explored.
This point is addressed in the new paragraph in the Discussion of the revised version.

Other points:
8. It would be good to highlight in a fig like fig 1a where the system is operating on the effective (smoothed) firing rate response curve. The implication is that it is very close to 0.1 on the horizontal axis. Actual mean firing rates of real cortical neurons (5-10 per second) are much less than maximum rates (a few hundred per second), which normally would place them to the left of this point in Fig 1a. I note though that the maximum possible firing rate is not clear from this figure – it would be good to mention it – so this comment is subject to that proviso.

We are not sure we fully understand this remark. For vanishing h, a rate of the order of 5Hz is compatible with the rate shown in fig.1a, for instance for h=10^-3. We are interested in the behaviour close to w_0c, therefore we did not explore the limit of very large rates.

9. Please clarify the units of s, h, w, f, etc. If they are dimensionless, the way in which they have been nondimensionalized needs to be made explicit. Are the numerical values consistent with independent measurements of these quantities? A referenced table of the assumed values of all quantities would be useful.

In Eq.1 the value s=0 corresponds to the membrane potential equal to the firing threshold -55mV. Since a is an a-dimensional number, w is also in mV, as the external field h. Therefore, s represents the distance of the membrane potential from the firing threshold in mV. In Eq.2 s is made a-dimensional by dividing by 1 mV. This notation was originally introduced by Benayoun et al in the definition of the model (ref.17). Conversely, the activation function f in Eq.2 and beta have both the dimension of an inverse time, ms^-1.

In the revised version we discuss the dimensions of the parameters after Eq.2, lines 126-127.

10. How robust are the results with respect to changes in parameters? This needs to be explored to reassure the reader that the results are more than a fluke.

We vary the system size (from 10^3 to 10^7), the unbalance in synaptic strengths (from 0 to 1) and the external field (from 10^-3 to 10^-6). All other parameters are not relevant to determine the critical behaviour.

11. Real cortical neurons receive around 10^5 spikes per second (10 per second via 10^4 synapses), so they produce 1 output spike for roughly each M=1000 input spikes for alpha = 0.1/ms (10 ms integration time). Hence, the input signal is essentially continuous, with some fluctuations, not spiky. Many spiking neuron simulations in the literature do not satisfy the requirement M>>1. Please evaluate and mention.

Our data concern the rate averaged over the entire population. Moreover, due to the all to all connectivity, all neurons can send inputs to each neuron, therefore in our case M>>1. The average rate can indeed exhibit different behaviours, from continuous to spiky, as shown in fig.3.

Response to Report of Referee 3

We thank the referee for writing that our " ... paper is interesting and on a timely topic with a long-lasting debate surrounding it". In the following we consider all comments raised by the referee.

Big Comment 1: One of the more commonly used aspects of critical phenomena in modern system neuroscience is the "crackling noise" scaling relation, which relates the average avalanche duration to the avalanche size according to a third scaling exponent. This has been used by many prominent studies to make claims about criticality: Friedman et al PRL 2012; Shew et al Nature Phys 2015; Ma et al Neuron 2019 and others. Does the stochastic WCM also conform to this scaling law? In my view, adding this to the current paper would make it substantially stronger and more related to the state of the art in this field. Moreover, this would be a much more interesting addition to the paper than the current diversion about different ways of defining avalanches (time bins vs. time series thresholding), which could be moved to methods or supplementary material.

We really thank the referee for this interesting suggestion. We are of course well aware of the attention that the Sethna scaling has attracted in the community. We have performed the analysis suggested by the referee on our system, i.e. a fully connected network with 50% inhibitory neurons. As noticed by the referee, Gillespie simulations allow to explore very large system sizes and therefore we are able to analyze a wide range of avalanche sizes. Results evidence the expected scaling behavior of the avalanche size vs. its duration: The gamma exponent is slightly larger than 2 (gamma~2.05), the value predicted by the Sethna scaling, for short avalanches (see new Fig.**5** in the manuscript). Moreover, the avalanche shapes for different sizes collapse onto a universal function for durations up to 400 time steps, corresponding to the scaling regime for the size distribution. However, the shape is strongly asymmetrical for all avalanche durations, i.e. non parabolic. We have confirmed this behaviour also for a different percentage of inhibitory neurons, 20 percent. At this point, a careful study in terms of system dimensionality and connectivity networks is required to fully understand this behavior. We plan to clarify this issue in the near future.

In the revised version we have added a novel paragraph (lines 282-297) and the new figure 5 to discuss the shape of avalanche and moved to the Methods section the discussion on the different avalanche definition.

Big Comment 2: Building on Big Comment 1, in my view the large digression (Figs 5-7) about how to define avalanches is not a very interesting addition to the paper. This disrupts the flow of the paper and it is very easy for a reader to lose interest. Perhaps it would be better to combine Figs 5-7 into one figure. Maybe even move it to the methods section or supplementary materials.
We have followed the referee suggestion. **In the revised version we have moved this discussion to the Methods section.**

Big Comment 3: The section titled "Requirements to assess critical behavior" is difficult to follow for someone who is not an expert on statistical physics of critical phenomena. As a step towards improving understandability, I suggest that the authors stick with a more explicit description of the Ising model and how it relates to assessing critical behavior. This would allow the authors to state more clearly what an order parameter is (magnetization), and what a control parameter is (temperature), and what an "external field" is, etc. By making it more specific, less general, the reader has a better chance of following along.

We thank the referee for the useful suggestion**. In the revised version we have added a new paragraph (lines 66-84) applying the concepts discussed for second order phase transitions in general to the specific case of the Ising model.**

Medium Comment 1: Considering that omega_0 is your control parameter, it would be helpful to provide more interpretation of the meaning of omega_0. I guess it represents a sort of e/i imbalance? It would also be helpful if the authors remind the reader of this meaning in the figure captions (maybe even in the axes labels of the plots).

This is indeed an interesting remark. The referee is right, w_0 is a parameter controlling the balance in synaptic strengths of excitatory and inhibitory populations. Interestingly, the critical value of this parameter, which plays the role of the temperature in the Ising model, is the ration of the dis-activation and activation rates for a single neuron. It therefore expresses the balance activity/quiescence at the cell level, which can be also view as a different form of E-I balance.
In the revised version we discuss more clearly the E-I balance features related to w_0 and its critical value at the beginning of the "Critical point" section (lines 168-174) and we specify in the figure caption of Fig.3 that the regimes obtained for different w_0 are critical and E-dominated.

Medium comment 2: When discussing and introducing the different alternatives for defining avalanches (time bins vs. time series threshold) it would be appropriate to cite some of the original experimental uses of these methods. For instance, Beggs & Plenz J Neurosci 2003 were among the first (maybe the first?) to use the time bin method. And Gautam et al PLos Comp Biol 2015 were among the first to use the time series thresholding methods.
We thank the referee for reminding us of these references. **In the revised version these two articles are now cited, Refs.[9,40].**

Small comments

Line 11: what is a "front of independent neurons". Consider rewording
The sentence refers to the general branching process. It should rather read as "front of independent sites that can either trigger further activity or die out". **We have corrected neurons into sites.**

Line 215: Should cite some relevant experiments
Unfortunately, we cannot cite here experimental data with a scaling regime of seven decades. To our knowledge, it is the first time that this effect is reported in the literature, which has been possible since Gillespie simulations allow to study systems with 10^7 neurons. This effect, the independence of the exponent values of the bin size, is novel and can be observed only for very large avalanche sizes.

In the revised version we cite at line 452-454 the main experimental references about scaling for experimental avalanches.

Fig 3 caption: Either get rid of the panel labels A-F or mention them in the caption
The caption has been changed accordingly.

Fig 3 caption: typo: single
Corrected.
